# Suppression of *OsVPE3* Enhances Salt Tolerance by Attenuating Vacuole Rupture during Programmed Cell Death and Affects Stomata Development in Rice

**DOI:** 10.1186/s12284-016-0138-x

**Published:** 2016-11-29

**Authors:** Wenyun Lu, Minjuan Deng, Fu Guo, Mingqiang Wang, Zhanghui Zeng, Ning Han, Yinong Yang, Muyuan Zhu, Hongwu Bian

**Affiliations:** 1 Institute of Genetics and Regenerative Biology, Key Laboratory for Cell and Gene Engineering of Zhejiang Province, College of Life Sciences, Zhejiang University, Hangzhou, China; 2 State Key Laboratory Breeding Base for Zhejiang Sustainable Pest and Disease Control, Institute of Virology and Biotechnology, Zhejiang Academy of Agricultural Sciences, Hangzhou, China; 3 Department of Plant Pathology and Huck Institute of Life Sciences, Pennsylvania State University, University Park, PA 16802 USA

**Keywords:** *OsVPE3*, Programmed cell death, Rice, Salt stress, Stomata, Vacuolar processing enzyme

## Abstract

**Background:**

Vacuolar processing enzymes (VPEs) are cysteine proteinases that act as crucial mediators of programmed cell death (PCD) in plants. In rice, however, the role of VPEs in abiotic stress-induced PCD remains largely unknown. In this study, we generated *OsVPE3* overexpression and suppression transgenic lines to elucidate the function of this gene in rice.

**Results:**

Survival rate and chlorophyll retention analyses showed that suppression of *OsVPE3* clearly enhanced salt stress tolerance in transgenic rice compared with wild type. Furthermore, fragmentation of genomic DNA was inhibited in plants with down-regulated *OsVPE3*. Vital staining studies indicated that vacuole rupture occurred prior to plasma membrane collapse during salt-induced PCD. Notably, overexpression of *OsVPE3* promoted vacuole rupture, whereas suppression of *OsVPE3* attenuated or delayed the disintegration of vacuolar membranes. Moreover, we found that suppression of *OsVPE3* caused decreased leaf width and guard cell length in rice.

**Conclusions:**

Taken together, these results indicated that suppression of *OsVPE3* enhances salt tolerance by attenuating vacuole rupture during PCD. Therefore, we concluded that *OsVPE3* plays a crucial role in vacuole-mediated PCD and in stomatal development in rice.

**Electronic supplementary material:**

The online version of this article (doi:10.1186/s12284-016-0138-x) contains supplementary material, which is available to authorized users.

## Background

Rice (*Oryza sativa* L.) is one of the most important cereal crops for more than half of the world’s population. According to estimates, the world will need to produce 25% more rice by 2030 to meet the challenges of feeding increasing populations. However, the increasing soil salinization of limited farmland is becoming a serious global threat to sustained rice production (Khatun and Flowers 1995; Sahi et al. 2006; Gao et al. 2007). Therefore, enhancing salt-tolerance is a serious concern for crop breeding programs.

Programmed cell death (PCD) is a highly conserved and genetically controlled process in multicellular organisms. PCD is involved in maintaining cellular homeostasis, development and senescence(Azeez et al. 2007; Williams and Dickman 2008), and this process is triggered by a variety of abiotic and biotic stresses (Huh et al. 2002; Lam 2004; Gechev et al. 2006). Plant and animal cells share many hallmarks of PCD, including cytoplasm shrinkage, chromatin condensation, DNA cleavage, mitochondrial swelling, organelle disruption and plasma membrane collapse (Mittler et al. 1997; van Doorn 2011; De Pinto et al. 2012). However, plants also exhibit unique features of PCD due to the presence of chloroplasts and vacuoles (Samuilov et al. 2003; Hatsugai et al. 2006; Kim et al. 2012; Wituszynska et al. 2015). Vacuoles are storage organelles that function as reservoirs for both hydrolytic enzymes and defence proteins, and vacuoles play several roles in stress response, development and pathogen defence. Recent studies have suggested that vacuole-mediated cell death is a response to various stresses in plants (Hatsugai et al. 2006; Hatsugai et al. 2015). Under salt stress, early events include the production of reactive oxygen species (ROS) and increased cytoplasmic calcium concentrations (Dionisio-Sese and Tobita 1998; Menezes-Benavente et al. 2004; Kudla et al. 2010) followed by PCD. In addition, vacuole rupture is a trigger for nuclear degradation during PCD (Obara et al. 2001). However, the genes and regulatory networks involved in vacuole-mediated cell death remain unidentified.

Vacuolar processing enzymes (VPEs) are cysteine proteinases involved in the processing of vacuolar proteins and the maturation of seed storage proteins in plants (Haranishimura et al. 1991; Hiraiwa et al. 1993; Rojo et al. 2003; Wang et al. 2009). VPEs are expressed in senescent tissues, and their expression patterns have been linked to PCD (Hara-Nishimura et al. 1998; Kinoshita et al. 1999). For example, it has been shown that VPEs regulate systematic cell death induced by viral, aluminium and heat stress, which is mediated by caspase-1-like activity during PCD (Hatsugai et al. 2004b; Li et al. 2012; Kariya et al. 2013). VPEs and caspase-1 share conserved structural properties, particularly the Asp pocket of caspase-1, which includes three crucial amino acids (Arg-179, Arg-341 and Ser-347) (Hatsugai et al. 2006). Although VPEs and caspase-1 share many similarities, the subcellular localizations of these two proteases are completely different. Namely, VPEs localize to vacuoles, whereas animal caspases localize to the cytosol. As initiators of plant PCD, VPEs trigger cell death through vacuolar collapse (Hatsugai et al. 2004a). To date, several types of VPEs have been reported to be involved in PCD as follows: NbVPE1a and NbVPE1b in Nicotiana; and AtγVPE in Arabidopsis. In rice, however, the role of VPEs in the vacuole-mediated cell death remains unknown.

The rice genome contains four *VPE* homologous genes as follows: *OsVPE1* (Os04g45470), *OsVPE2* (Os01g37910), *OsVPE3* (Os02g43010) and *OsVPE4* (Os05g51570) (Deng et al. 2011). Phylogenetically, *OsVPE1* and *OsVPE3* are more similar to Arabidopsis *At*β*VPE*, whereas *OsVPE2* and *OsVPE4* are more similar to *At*α*VPE* and *At*γ*VPE. OsVPE1*, a homolog of *At*β*VPE*, plays a crucial role in the maturation of glutelins in seeds (Wang et al. 2009). Previous work by our group has shown that the transcription of *OsVPE2* and *OsVPE3*, but not of *OsVPE1* and *OsVPE4*, can significantly enhance salt-induced PCD (Deng et al. 2011; Kim et al. 2014). In this study, we aimed to elucidate the role of *OsVPE3* in the context of salt stress. To determine the function of *OsVPE3* in rice under salt stress, we generated transgenic lines to either overexpress and suppress *OsVPE3*, and we found that *OsVPE3* regulates the collapse of vacuolar membranes during PCD. In addition, we found that *OsVPE3* plays a role in leaf and stomata development in rice.

## Results

### Expression pattern and localization of OsVPE3

**Fig. 1:**
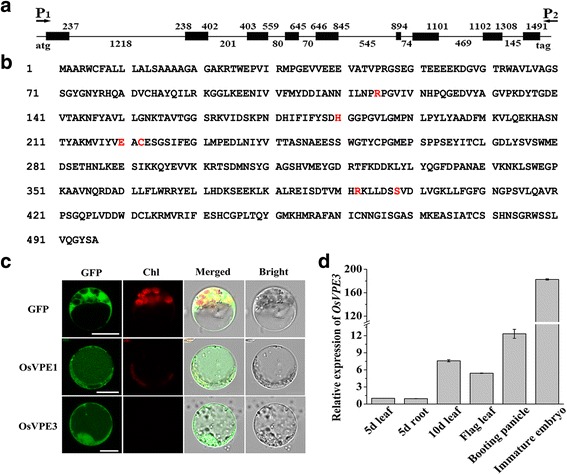
Sequence analysis, subcellular localization and expression pattern of OsVPE3. **a** The *OsVPE3* gene structure based on the genomic sequence and full-length cDNA clone (BAC41387). Black boxes represent exons. Arrows indicate the primers used to isolate *OsVPE3*cDNA. **b** OsVPE3 amino acid sequence. Amino acids necessary for caspase-like activity are indicated by coloured letters. **c** The subcellular localization of OsVPE1 and OsVPE3 by confocal fluorescent microscopy. Scale bar = 10 μm. **d** qRT-PCR analysis of *OsVPE3* expression in various rice tissues. Total RNA was extracted from leaf, root, booting panicle and immature embryo samples. *OsUBQ5* was used as an internal control. Values are means, and error bars represent the SD from three independent experiments

A comparison between genomic DNA and cDNA revealed that*OsVPE3* (Os02g43010) contains nine exons and eight introns (Fig. 1a), and *OsVPE3* encodes a peptide of 496 amino acids. The red letters in Fig. 1 indicate essential amino acids in the catalytic site and substrate pocket that mediate caspase-like activity (Fig. 1b) (Nicholson 1999; Hara-Nishimura et al. 2005; Hatsugai et al. 2015).

To determine the subcellular localization of *OsVPE3*, the full-length cDNA was fused to green fluorescent protein (GFP) under control of the 35S promoter. An *OsVPE1-GFP* fusion gene driven by the 35S promoter was used as a control. OsVPE1, a seed-type vacuolar processing enzyme, has been shown to localize to vacuoles in onion cells (Wang et al. 2009). These constructs were transiently transformed into rice protoplasts. We searched for GFP signals in vacuoles for the *35S*:*OsVPE1-GFP* and *35S*:*OsVPE3-GFP* fusion constructs, and no GFP signal was observed in vacuoles for the *35S*: *GFP* construct (Fig. 1c). It was reported that GFP was unstable in the acidic vacuoles of plants under the light condition, because light would cause the rapid degradation of 27-kDa GFP by pre-existing proteinase in vacuoles at pH5.5 (Tamura et al., 2003). In protoplast transient assay, isolated protoplasts were kept in the dark condition to protect from light damage, thus the GFP fluorescence can be observed in the vacuoles..Consequently, this result suggested that OsVPE3 is a vacuole-targeting protein similar to OsVPE1.

To investigate the expression pattern of *OsVPE3* in rice, transcription levels were examined in various tissues by quantitative RT-PCR. Expression analysis revealed that *OsVPE3* is expressed in tissues of the leaf, root, booting panicle and immature embryo (Fig. 1d). *OsVPE3* expression increased rapidly in the leaves of 10-day-old seedlings compared with 5-day-old seedlings. The highest transcriptional expression level of *OsVPE3* was detected in immature embryos. These results demonstrated that *OsVPE3* is actively transcribed in the leaves and developing embryos of rice.

### Generation of Transgenic Rice Lines

**Fig. 2:**
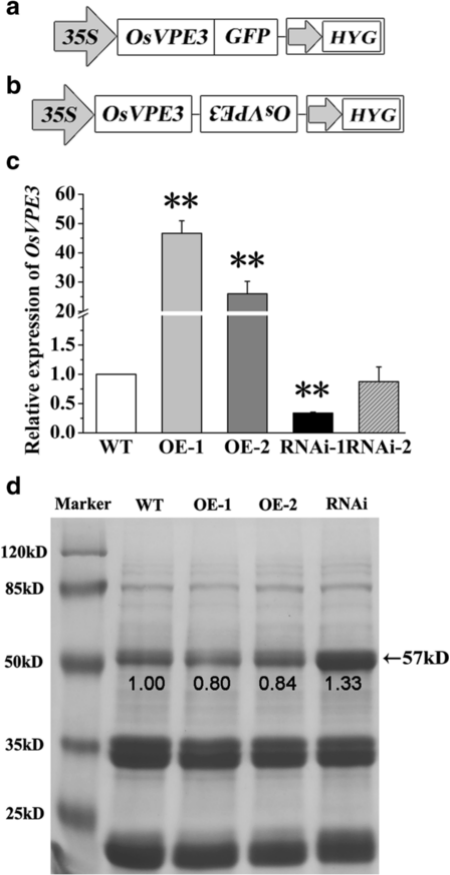
Generation and analysis of transgenic lines. A diagram of the vector constructs used to generate the overexpression lines (**a**) and RNAi line (**b**). **c** Expression levels of *OsVPE3* in WT and transgenic lines were determined by qRT-PCR. *OsUBQ5* was used as an internal control. Values are means, and error bars represent the SD (*n* > 1) from three independent experiments. Asterisks indicate a significant difference between WT and transgenic lines (t-test; **, *P* < 0.01). **d** SDS-PAGE analysis of seed storage proteins in WT and transgenic lines. The intensity of protein bands was measured by Image J software. Arrow indicates the 57-kDa glutelin precursor

In our previous work, we found that the expression of *OsVPE3* is dramatically induced by salt stress (Deng et al. 2011; Kim et al. 2014). However, the role of the *OsVPE3* gene in rice remains largely unknown. To investigate if *OsVPE3* is involved in the process of PCD, *OsVPE3* was overexpressed and suppressed using transgenic lines (Fig. 2a, b). Homozygous T3 transgenic seeds were used for further investigation. Quantitative RT-PCR analysis confirmed that the expression levels of *OsVPE3* were strongly increased in the overexpression lines (OE-1 and OE-2) and decreased significantly in the RNA interference transgenic lines (RNAi-1) compared with WT (Fig. 2c).

Previous studies showed that *OsVPE1* plays a crucial role in the maturation of glutelins in seeds (Wang et al. 2009). To examine the role of *OsVPE3* on seed proteins, we performed SDS-PAGE analysis to compare the proteins in WT and transgenic lines. Our data showed that the major storage proteins yielded three bands of approximately 20, 40 and 57 kDa in rice (Fig. 2d). The 57-kDa protein is a glutelin precursor (Yamagata et al. 1982; Krishnan and Okita 1986). Compared with WT, 57 kDa protein levels was increased to 1.33 fold in the *OsVPE3-*RNAi line, whereas reduced to approximately 0.80-0.84 in the overexpression lines.(Fig. 2d). Accordingly, the grain width and 1000-grian weight of the RNAi line decreased significantly compared with WT and overexpression lines (Additional file 1: Figure S1). These results indicated that *OsVPE3* is involved in the processing of seed storage proteins in rice.

### Suppression of OsVPE3 Enhances Salt Stress Tolerance in Rice

**Fig. 3:**
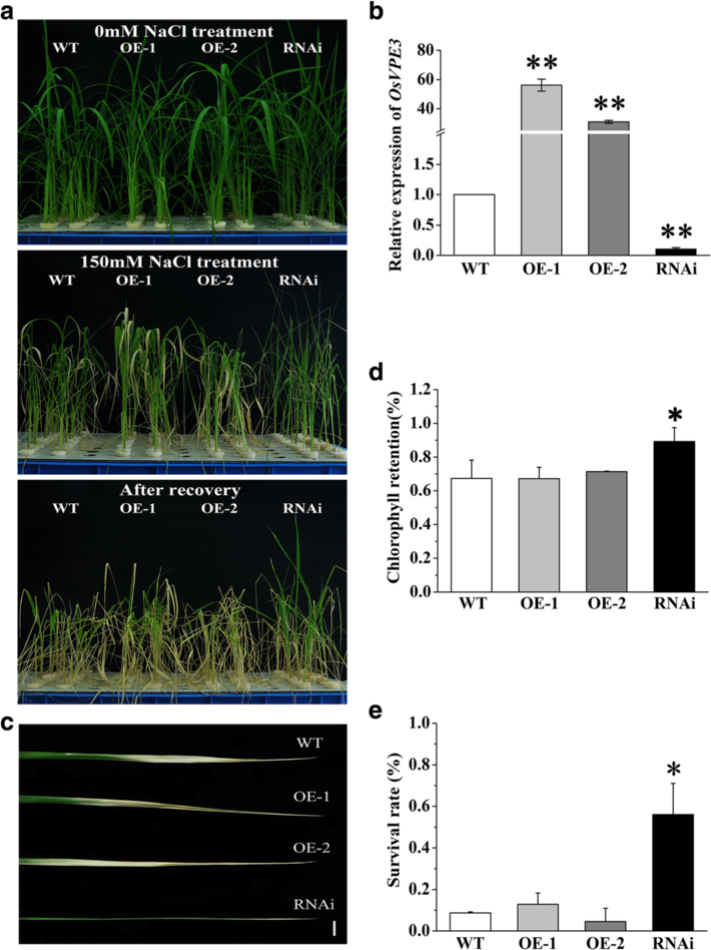
Suppression of *OsVPE3* improved survival rate and inhibited chlorosis under salt stress. **a** Plants were grown for 4 weeks, treated with 150 mMNaCl for 3 days and then allowed to recover for 7 days. **b** Expression levels of *OsVPE3* in WT and transgenic lines as determined by qRT-PCR. Total RNA was extracted from the fourth leaves. *OsUBQ5* was used as an internal control. Values are means, and error bars represent the SD from three independent experiments. Asterisks indicate a significant difference between WT and transgenic lines (t-test; **, *P* < 0.01). **c** The fourth leaves of NaCl-treated plants. Bar = 1 cm. **d** Chlorophyll retention in the fourth leaves of NaCl-treated plants prior to recovery. Values are means, and error bars represent the SD from three independent experiments. Asterisks indicate a significant difference between WT and transgenic lines (t-test; *, *P* < 0.05). **e** Survival rates of NaCl-treated plants 7 days after recovery. Values are means, and error bars represent the SD (*n* > 20) of three independent experiments. Asterisks indicate a significant difference between WT and transgenic lines (t-test; *, *P* < 0.05)

Based on our previous studies, we hypothesized that *OsVPE3* might act as a trigger in salt-induced PCD. To determine if *OsVPE3* is involved in salt stress tolerance in rice, 4-week-old WT and transgenic plants were treated with 150 mM NaCl for 3 days. Before NaCl treatment, the expression levels of *OsVPE3* were measured in the fourth leaf of each line, confirming that the transcript levels of *OsVPE3* were increased in overexpression lines (OE-1 and OE-2) and significantly suppressed in the RNAi transgenic lines (Fig. 3a, b). Under salt stress, chlorosis is a common symptom in rice leaves. Chlorophyll contents were measured after 3 days of NaCl treatment, and then survival rates were calculated after 7 days of recovery culture under normal growth conditions. When the plants were exposed to 150 mM NaCl for 3 days, the leaves of WT and overexpression lines turned yellow. In contrast, the chlorosis phenotype was significantly weakened in the RNAi line (Fig. 3c, d). Approximately 56.1 ± 15.0% of RNAi plants remained alive after recovery culture, whereas the survival rates of WT and overexpression transgenic lines (OE-1, OE-2) were significantly lower than that of RNAi line (Fig. 3a, e). Remarkably, the RNAi line had a significantly higher chlorophyll content and relative survival rate compared with the WT and overexpression lines (Fig. 3d, e). These results demonstrated that suppression of *OsVPE3* inhibits the development of chlorosis and improves salt tolerance in rice.

### Suppression of OsVPE3 Inhibits the Formation of Dna Ladders in Salt-Induced PCD

**Fig. 4:**
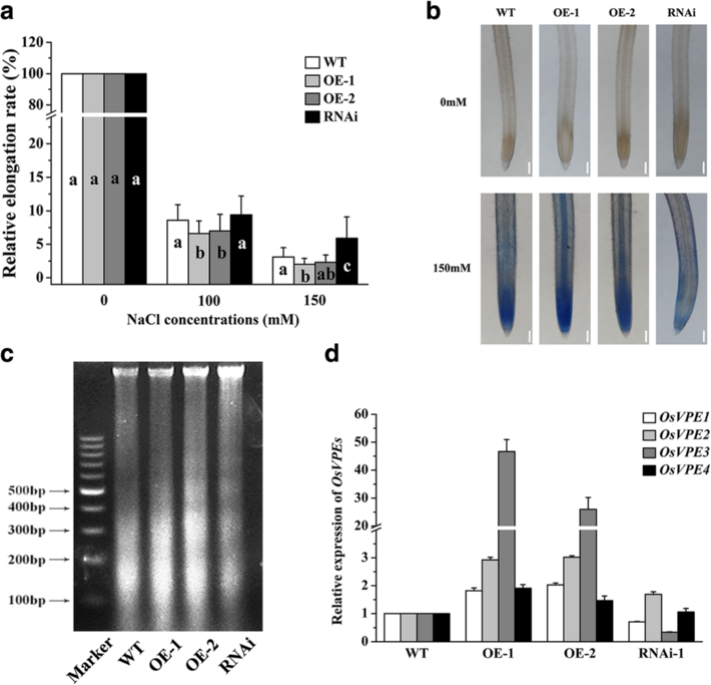
Inhibition of the primary root growth and DNA ladder formation in WT and transgenic lines under salt stress. **a** Three-day-old seedlings were treated with 0, 100 and 150 mM NaCl for 5 days. The root relative elongation rates were calculated as described in the Materials and methods. Values are means, and error bars represent the SD of three independent experiments *n* > 30) and the different letters denote the significant difference among different lines (ANOVA, *P* < 0.05). **b** Primary root cell viability was presented using Evans Blue dye in WT and transgenic lines treated with 0 or 150 mMNaCl for 3 days. Bars = 200 μm. **c** DNA ladder formation after treatment with 300 mMNaCl for 8 h in root tips. **d** Relative expression levels of *OsVPE* homology genes determined by qRT-PCR. Total RNA was extracted from the roots. *OsUBQ5* was used as an internal control. Values are means, and error bars represent the SD from three independent experiments

To further investigate the effect of *OsVPE3* on rice tolerance to salt stress*,* 3-day-old WT and transgenic seedlings were treated with high concentration NaCl (100 mM, 150 mM) for 3 days. The results showed that RNAi seedlings had the highest root relative elongation rate and overexpression seedlings were the most sensitive to salt stress (Fig. 4a). At the same time, Evans Blue staining confirmed that the root tips of the RNAi line maintained highest cell viability after exposure to 150 mM NaCl for 3 days compared to other lines (Fig. 4b). The fragmentation of genomic DNA is a typical biochemical and morphological feature of PCD. To determine if the suppression of *OsVPE3* can inhibit salt-induced PCD, DNA fragmentation was measured in transgenic lines and WT plants treated with 300 mM NaCl for 8 h. As shown in Fig. 4c, clear and visible DNA laddering was observed in the *OsVPE3*-overexpression lines, whereas DNA laddering was significantly inhibited in the RNAi line. This result suggested that suppression of *OsVPE3* inhibits the formation of DNA ladders during salt-induced PCD.

Because *OsVPE3* has high homology with *OsVPE1*, we performed qRT-PCR experiment to detect the expression levels of *OsVPE* homologous genes in the roots of transgenic lines (Fig. 4d). The results showed that the expression level of *OsVPE3* was decreased dramatically in RNAi line; meanwhile, *OsVPE1* expression was also reduced compared with the WT. The expression level of *OsVPE3* was much more decreased than *OsVPE1* in the RNAi line*.* Our previous work has shown that the transcription of *OsVPE2* and *OsVPE3*, but not of *OsVPE1* and *OsVPE4*, can significantly increase in the salt-induced PCD (Deng et al. 2011; Kim et al. 2014), suggesting that the transcription of *OsVPE1* was not responsive to salt stress in rice roots (Deng et al. 2011). Therefore, we conclude that *OsVPE3* plays a crucial role in salt stress-mediated PCD.

### Suppression of OsVPE3 Helps Maintain the Integrity of Vacuolar Membranes During Salt-Induced Pcd

**Fig. 5:**
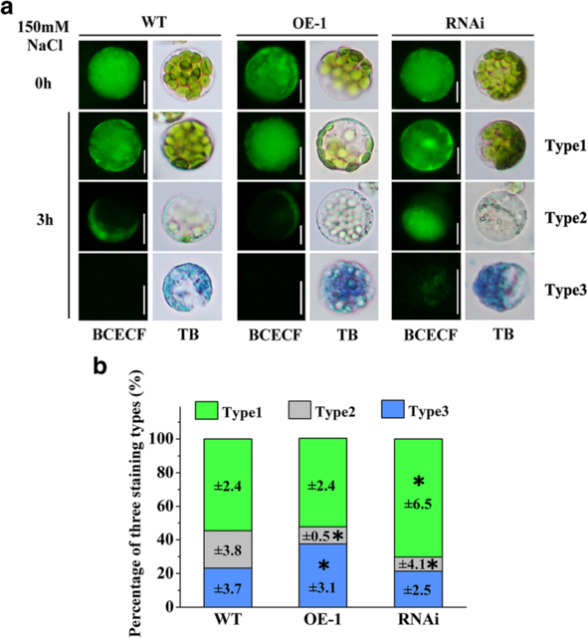
Suppression of *OsVPE3* alleviates vacuole rupture under salt stress. **a** Protoplasts from WT and transgenic lines were stained with the BCECF-AM vital dye (final concentration = 10 μM) for 2 h and then treated with 100 mM NaCl for 3 h. Before the BCECF fluorescent images were inspected, the protoplasts were stained with Trypan Blue (TB). Type 1 protoplasts were living cells with intact vacuoles. Type 2 protoplasts were living cells with disintegrated vacuolar membranes. Type 3 protoplasts were dead. **b** Statistical analysis of three types of protoplasts after treatment with 100 mM NaCl. Data represent the means ± SD (*n* > 100) for three independent replicates. Asterisks indicate a significant difference between the WT and transgenic lines (t-test; *, *P* < 0.05)

Unlike in animal cells, the vacuole is a unique organelle used to store a variety of hydrolytic enzymes and defence proteins in plant cells (Neuhaus et al. 1991; Yamada et al. 2001). The rupture of vacuolar membranes releases these components and leads to cell death (Mino et al. 2006). To elucidate the role of *OsVPE3* in the integrity of vacuolar membranes during PCD, rice protoplasts were stained with Trypan Blue and the BCECF-AM fluorescent probe. Trypan Blue staining is a reporter of cell death based on disintegrated cellular membranes, and BCECF-AM labels the vacuole lumen as green fluorescence to reveal intact vacuoles (Swanson and Jones 1996). BCECF-staining revealed that protoplasts prior to NaCl treatment accumulated strong fluorescence signal in vacuoles in both WT and transgenic lines (Fig. 5a, the first horizontal low). Protoplasts subjected to 100 mM NaCl treatment for 3 h can be sorted into three types as follows (Fig. 5a, b): Type 1, BCECF-positive and Trypan Blue-negative, indicating living cells with intact cellular membranes and vacuoles; Type 2, BCECF-negative and Trypan Blue-negative, indicating living cells with disintegrated vacuolar membranes (as shown by distribution of BCECF fluorescence signal outside of the vacuoles); and Type 3, BCECF-negative and Trypan Blue-positive, indicating dead cells with collapsed vacuoles (as shown by no BCECF fluorescence signal). After 100 mM NaCl treatment, the survival protoplasts (type 1) decreased to 54.6 ± 2.4 and 52.7 ± 2.4% of the levels in the WT and OE-1 lines, respectively. In contrast, the survival rate of protoplasts (Type 1) from the RNAi line was 70.3 ± 6.5%, which was significantly higher than that of the WT and OE-1 lines. The results showing that many of the protoplasts (Type 2) with degraded vacuolar membranes were alive suggested that cell death was preceded by vacuolar collapse. Only 8.4 ± 4.1% of the RNAi cells belonged to Type 2, which involved vacuole rupture before cell death. Interestingly, 37.5 ± 3.1% of *OsVPE3-*overexpressing cells belonged to Type 3, which was significantly higher than in the WT and RNAi lines. Notably, certain Type 3 protoplasts from the RNAi line still showed weak fluorescence in the vacuolar region, thereby suggesting that *OsVPE3*-RNAi attenuated or delayed the disintegration of vacuolar membranes even after cell death. Taken together, these results demonstrated that suppression of *OsVPE3* prevents vacuole rupture during salt-induced PCD (Fig. 5a).

### Suppression of OsVPE3 Results in Decreased Leaf Width and Stomatal Size

**Fig. 6:**
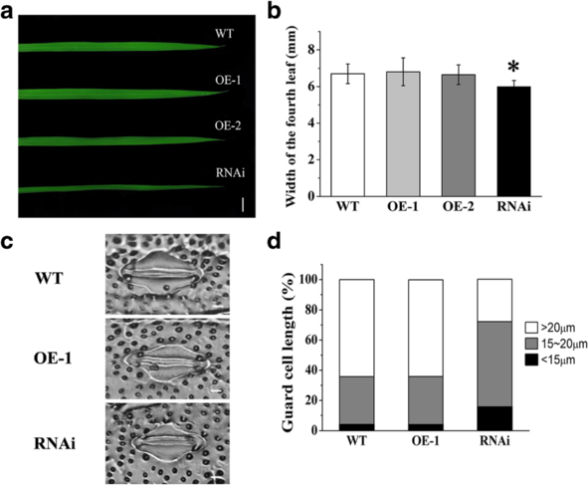
Fourth leaf width and guard cell size in WT and transgenic lines. **a** Images of fourth leaves of WT and transgenic lines. Bars = 1 cm. **b** Measurements of the maximum fourth leaf width in WT and transgenic lines. Values indicate the mean, and error bars represent the SD (*n* >10). An asterisk indicates a significant difference between the WT and transgenic lines (t-test; *P* < 0.05). **c** Images of stomata in WT and transgenic lines. Bars = 5 μm. **d** Percentage of stomata with various guard cell lengths in WT and transgenic lines (*n* > 500)

Compared with WT, there were no obviously morphological changes in the transgenic plants at the early seedling stage. However, after four weeks of growth, the RNAi transgenic line exhibited narrower leaves than WT (Fig. 6a). Blade width of the fourth leaf was approximately 6.00 ± 0.54 cm in the WT plants, whereas the blade width was approximately 5.50 ± 0.33 cm in the RNAi line (Fig. 6b). Further observation revealed stomata size in the RNAi line was smaller than in WT plants (Fig. 6c). Statistical analysis showed that only 25% of guard cells were longer than 20 μm in the RNAi line whereas 64% in the WT plants. The majority of guard cells were between 15 and 20 μm in the RNAi line (Fig. 6d). There were no significant differences in leaf width and guard cell length between the OE-1 line and WT plants. The expression pattern of *OsVPE3* showed that *OsVPE3* is actively transcribed in the leaves and developing embryos of rice (Fig. 1d). We speculated that the expression levels of *OsVPE3* were abundant enough for the leaf development in WT, thus the *OsVPE3*-overexpression lines exhibited weak phenotype compared with the WT.

In addition, we tested the percentage of water loss of detached leaves from WT and transgenic lines. The data indicated that the percentage of water loss of RNAi leaves was significantly lower than other lines (Additional file 1: Fig. S2), suggesting suppression of *OsVPE3* enhanced the dehydration tolerance. The above results demonstrated that suppression of *OsVPE3* caused decreased leaf width and guard cell length, promoting a dehydration tolerance in rice leaves.

**Fig. 7:**
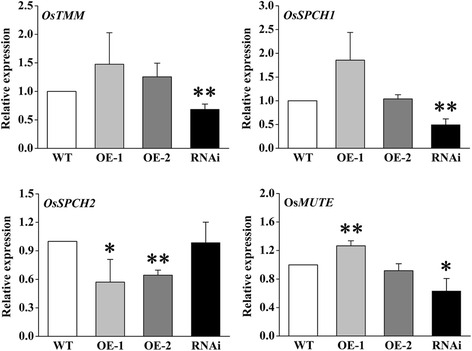
Effect of *OsVPE3* on the expression levels of genes related to stomatal development. Expression levels of *OsTMM*, *OsSPCH1*, *OsSPCH2*, and *OsMUTE* in WT and transgenic lines as determined by qRT-PCR. Total RNA was extracted from the shoots of 5-day-old plants. *OsUBQ5* was used as an internal control. Values are means, and error bars represent the SD from three independent experiments. Asterisks indicate a significant difference between WT and transgenic lines (t-test; **, *P* < 0.01)

Furthermore, we analysed the expression of genes related to stomatal development by qRT-PCR. According to known reports, there are five critical genes related to the development of stomata in rice, namely *OsTMM* (*TOO MANY MOUTHS*), *OsSPCH1* (*SPEECHLESS*), *OsSPCH2*, *OsMUTE* and *OsFAMA* (Liu et al. 2009). *OsTMM* is a receptor for extracellular ligand, characterized as a set of key regulators in stomatal production and patterning (Balcerowicz and Hoecker 2014). *OsSPCH1*, *OsSPCH2*, *OsMUTE* and *OsFAMA* are essential transcription factors in the stomata patterning and development (Liu et al. 2009). We monitored the expressions of these genes (the data of *OsFAMA* is not shown because of its low expression level in rice leaves). As shown in Fig. 7, the expression levels of *OsTMM*, *OsSPCH1* and *OsMUTE* were significantly down-regulated in the RNAi line compared with WT. Consistently, these three genes showed up-regulated tendencies in overexpression lines, while only the expression of *OsSPCH2* decreased significantly. It revealed that *OsVPE3* might mainly affect the level expression of *OsTMM, OsSPCH1* and *OsMUTE* in stomata developmental pathway to change the guard cell size.

## Discussion

The main purpose of this study was to understand the role of *OsVPE3* in vacuole-mediated PCD following salt stress in rice. *OsVPE3* overexpression and suppression transgenic lines were created to elucidate the function of *OsVPE3* in rice. Our results demonstrated that *OsVPE3* plays a crucial role in the salt stress response by regulating the collapse of vacuolar membranes during PCD. In addition, *OsVPE3* affected leaf and stomata guard cell development in rice.

High salinity is one of the most important abiotic stresses during crop breeding. High salinity activates the salt-overly-sensitive (SOS) system in plants, which leads to sodium exclusion from the cytosol (Zhu 2003). Under high salt conditions, excess Na^+^ accumulates in the plant, thus increasing influx of Na^+^ and efflux of K^+^(Serrano and Rodriguez-Navarro 2001; Horie et al. 2012). It has been proposed that these changes can decrease the cytosolic K/Na ratio, thus elevating concentrations of the intracellular second messenger Ca^2+^ (Kudla et al. 2010) and causing ROS bursts (Zhu 2001). As a consequence of increased K/Na ratios and salinity-induced ROS, programmed cell death would be finally triggered (Huh et al. 2002; Lin et al. 2006; Shabala 2009). A recent study in rice by our group revealed that overexpression of BCL-2, an anti-apoptotic protein, significantly reduces NaCl-induced K+ efflux and represses the expression of VPEs, thereby alleviating PCD symptoms(Deng et al. 2011; Kim et al. 2014).

Salt-induced PCD in plants and animals shares many consequences, including DNA fragmentation, nuclear condensation, nuclear deformation, mitochondrial involvement and endonuclease activity (Li et al. 2007; Jiang et al. 2008). Analysis of DNA laddering indicated that PCD occurs in rice seedlings under salt treatment (Fig. 4c). Compared with WT, overexpression of *OsVPE3* strongly enhanced genomic DNA fragmentation, whereas *OsVPE3* interference strongly repressed DNA fragmentation during PCD. This finding suggested that *OsVPE3* likely plays a crucial role in the salt-induced PCD in rice.

The VPE family was originally identified as a group of processing enzymes responsible for the maturation of seed storage proteins in protein storage vacuoles (Haranishimura et al. 1991; Haranishimura et al. 1993). It was originally reported that NtVPEs have caspase-1 activity and are essential for the virus-induced hypersensitive response involving PCD, which led to the proposition of a new cell death mechanism mediated by VPE and cellular vacuoles (Hatsugai et al. 2004b). Subsequently, a similar function was proposed for AtγVPE in mycotoxin-induced cell death (Kuroyanagi et al. 2005). Recently, further studies have shown that VPEs are also involved in cell death under abiotic stresses (Zhang et al. 2013b). For example, AtγVPE, which is mediated by MPK6, affects heat-shock-induced PCD in Arabidopsis (Li et al. 2012). Our study found that *OsVPE3* regulated salt-induced PCD in rice.

Vacuoles are essential organelles in plants, which have multiple functions including storage of a wide variety of ions, proteins and other metabolites, and maintain cytosolic ion homeostasis (Boller and Wiemken 1986; Rea and Sanders 1987). Plant vacuoles also play critical roles in stress responses, development and pathogen defence. There have been many reports concerning the relationship between vacuoles and cell death (Hara-Nishimura and Hatsugai 2011; Higaki et al. 2011). Vacuole rupture triggers nuclear degradation during PCD (Obara et al. 2001). The discovery of VPE functions in vacuoles could further explain the molecular mechanism of vacuole-mediated PCD. Similar to characterized OsVPE1, OsVPE3 localized to vacuoles (Fig. 1c). We found that vacuole integrity was impaired prior to cell death under salt treatment in WT plants. However, suppression of *OsVPE3* markedly enhanced the maintenance of vacuolar membranes even in the context of cell death. In contrast, overexpression of *OsVPE3* accelerated the rupture of vacuole membranes (Fig. 5). Our results strongly supported the hypothesis that *OsVPE3* plays a crucial role in vacuole rupture during PCD. To date, the mechanism of VPE-mediated vacuole rupture remains unclear, due to a lack of evidence that the VPEs interact other vacuolar proteins. Based on the characteristics of VPEs, we propose that VPEs expression is upregulated by disturbances in ion homeostasis in response to abiotic stresses and that VPE precursors are self-activated to process vacuolar hydrolases and proteases, leading to vacuole rupture and cell death.

In addition to vacuole rupture, there is another interesting finding concerning the role of *OsVPE3* in the development of stomata in rice. The stoma is known to be an important structure for controlling gaseous exchange and water release by transpiration (Assmann 1993), and function under stress conditions (Zhang et al. 2013a). At the same time, stomata movement may be affected by VPEs in *Arabidopsis* (Albertini et al. 2014). We found that leaves in the RNAi line were more curled than in wild type and that this phenomenon was more apparent under salt stress (Fig. 3). Further research showed that stomata size in the RNAi line was smaller than in WT plants (Fig. 6c, d). Small stomata may alter transpiration to improve resistance in plants. Transcriptome analysis has revealed strong expression of *At*γ*VPE* in Arabidopsis guard cells (Albertini et al. 2014). qRT-PCR analysis has confirmed that γ*VPE* expression in guard cells is higher than in whole leaves, thus suggesting that this gene plays a critical role in guard cells (Albertini et al. 2014). Moreover, *Arabidopsis* γ*VPE* knockout mutants reduced stomata opening and increased resistance to desiccation (Albertini et al. 2014). Our data showed that suppression of *OsVPE3* down-regulated the expression levels of *OsTMM, OsSPCH1* and *OsMUTE* in the stomata developmental pathway, leading to affect guard cell size in the *OsVPE3*-RNAi line. This finding suggests that *OsVPE3* might play a role in stomata development in rice.

## Conclusions

Our results demonstrated that *OsVPE3* plays a crucial role in the salt stress-induced PCD by regulating the collapse of vacuolar membranes. In addition, *OsVPE3* affected leaf and stomata guard cell development in rice. These findings are relevant for enhancing salt-tolerance via genetic engineering in crop breeding programs.

## Methods

### Growth Conditions

Seeds were surface sterilized with 10% sodium hypochlorite (v/v) for 30 min, rinsed 5 times in deionized water and soaked in deionized water at 30 °C for 2 days in the dark. After germination, the seeds were transferred to a nutrient solution culture (Yoshida et al. 1976) at pH 5.0–6.0 in a greenhouse at 28 °C under a 16:8 h light: dark cycle. The 3-day-old seedlings were treated with 300 mM NaCl for DNA ladder detection. For the expression pattern analyses, tissues were collected at different stages following seedling growth under these conditions. After 4 weeks of growth, the plants were treated with various concentrations of NaCl.

### Constructs and Plant transformation

All wild-type and mutant transgenic lines were generated in the *Oryza sativa* L. *ssp. japonica cv. Nipponbare* rice background.

For the overexpression constructs, the cDNA sequence of *OsVPE3* (approximately 1488 bp) was amplified from the cDNA library of Nipponbare using gene-specific primer pairs. The fragments were then cloned into the pENTR⁄D-TOPO vector (Invitrogen, Carlsbad, CA, USA) and then into the destination vector (pH7FWG2.0) by LR clonase reactions.

For the RNA interference (RNAi) constructs, a 745-bp fragment was amplified from *OsVPE3*, inserted into the pENTR⁄D-TOPO vector (Invitrogen) and then cloned into pH7GWIWG2 (I) by LR clonase reactions.

Rice transformation was performed using the *Agrobacterium tumefaciens*-mediated co-cultivation method. Transformed calli were selected on hygromycin medium. T0 plants were self-pollinated over two generations to obtain homozygous T2 transgenic seeds. Homozygous T3 seeds were used in this study.

Primers used in this work are listed in Additional file 1: Table S1. The gene constructs used for rice transformation were verified by sequencing.

### Total RNA extraction and qRT-PCR Assay For Osvpe3 Expression

Total RNA was isolated using the TRIzol RNA extraction kit (Invitrogen), and first-strand cDNA was synthesized using the ReverTra Ace qPCR RT Master Mix with gDNA Remover for qRT-PCR (TOYOBO). Quantitative RT-PCR was performed using the Master cycler *ep realplex* system (Eppendorf, Hamburg, Germany) and the SYBR PrimeScript RT-PCR kit (Perfect Real Time; TaKaRa). *OsUBQ5* was amplified as a control for the template. All primers used in this work are listed in Additional file 1: Table S1.

### Protein extraction from rice grains and SDS-PAGE assays

Mature seeds harvested from T3 homozygous plants were used for protein extraction. The protein extraction was performed on the described methods (Takemoto et al. 2002), and the proteins were analysed by SDS-PAGE. The density of the protein bands was measured by using the Image J software.

### Subcellular Localization

The OsVPE1:GFP and OsVPE3:GFP fusions were created using Gateway cloning (Invitrogen). The full-length cDNAs of *OsVPE1* and *OsVPE3* (lacking stop codons) were amplified and inserted into the pENTR/D-TOPO vector (Invitrogen), and they were subsequently cloned into pUGW5 using LR clonase reactions. The fusion constructs were transformed into rice protoplasts using PEG-mediated transfection according to our previous study (Bai et al. 2014). Following transformation, rice protoplasts were incubated at 25 ± 2 °C in the dark for 12–16 h prior to observation with a Zeiss LSM710 NLO two-photon microscopy (Germany).

### Chlorophyll Content Assay

Four-week-old plants were treated with 150 mM NaCl for 3 days prior to measuring chlorophyll content. Chlorophyll was extracted from the tip of the fourth leaf. Briefly, each sample (0.1 g) was placed into a 10-mL tube containing 4 mL of extraction solution (1: 1 ethanol: acetone) and incubated in the dark for 24 h at 25 °C. Absorbance of the extracts at 663 and 645 nm was measured using a Spekol spectrocolorimeter (Carl Zeiss GmbH, Jena, Germany). Total chlorophyll content was calculated using the following formulas (Yu et al. 2006):

$$ \left[\mathrm{C}\mathrm{h}\mathrm{l}\ \mathrm{a}\right] = 12.7\times \mathrm{A}663 - 2.69 \times \mathrm{A}645 \times \mathrm{V}/\left(1000 \times \mathrm{W}\right) $$

$$ \left[\mathrm{C}\mathrm{h}\mathrm{l}\ \mathrm{b}\right] = 22.9 \times \mathrm{A}645 - 4.68 \times \mathrm{A}663 \times \mathrm{V}/\left(1000 \times \mathrm{W}\right) $$

$$ \left[\mathrm{C}\mathrm{h}\mathrm{l}\ \mathrm{a} + \mathrm{b}\right] = 20.2 \times \mathrm{A}645 + 8.02 \times \mathrm{A}663 \times \mathrm{V}/\left(1000 \times \mathrm{W}\right) $$

All experiments were repeated three times.

### Root Elongation Assay

After germination, sterilized seeds were sown on filter paper moistened with 0.1 mM CaCl_2_. Before treatment with NaCl, the primary root lengths of 3-day-old seedlings were measured and recorded as L_C0_ and L_T0_. After treatment with NaCl (0, 100, 150 mM) for 3 days, the primary root lengths were recorded as L_C3_ and L_T3_. Root relative elongation rates were calculated using the formula previously reported by Pan *et al*. (2004):

$$ \mathrm{R}\mathrm{E}\mathrm{R}\ \left(\%\right) = \left({\mathrm{L}}_{\mathrm{T}3} - {\mathrm{L}}_{\mathrm{T}0}\right)/\left({\mathrm{L}}_{\mathrm{C}3}\hbox{--}\ {\mathrm{L}}_{\mathrm{C}0}\right) \times 100\% $$

### Evans Blue Staining

The cell viability of primary root was evaluated using Evans Blue staining. After treatment with 0 or 150 mM NaCl for 3 days, 6-day-old seedlings were stained with 2.5% Evans Blue for 10 min and then washed twice with deionized water. Prior to observation, the seedlings were soaked in transparent agent for 12 h.

### DNA Ladder Analysis

After 8 h of treatment with 300 mMNaCl, the roots tips of the rice seedlings were collected and ground in liquid nitrogen. DNA was isolated using the CTAB method and then digested with 100 g/mL DNase-free RNase for 1 h at 37 °C to eliminate RNA contamination. For each sample, an aliquot of DNA (20 μg) was separated using a 2% (w/v) agarose gel, stained with 0.1 μg/mL ethidium bromide in TE buffer (10 mMTris-HCl, pH 8.0; 0.5 mM EDTA) and washed once with TE buffer. The fragmented DNA was observed under UV light using a photostation (UVI, Cambridge, UK).

### BCECF and Trypan Blue Staining

Protoplasts were incubated with 10 μM BCECF-AM (Molecular Probes, USA) for 2 h at 25 °C in the dark and then treated with 100 mM NaCl for 3 h. Prior to observation, protoplasts were washed twice with W5 solution and stained with 0.04% Trypan Blue for 3 min. BCECF signal was visualized with excitation at 465–495 nm and emission at 515–555 nm using a band pass filter based on a previous method (Tang et al. 2012).

### Leaf Water Loss Assay

Plants germinated under normal growth conditions for 4 weeks. The leaves were detached from various lines with same age and position, and weighed immediately as the initial fresh weight. They were then placed in clean filter papers, and incubated at 25 °C. The decreases in fresh weight were recorded at every 20 min for 5 h. Water loss was presented as percentage of fresh weight loss versus the initial fresh weight (Zhang et al. 2012).

### Stomata Observation

The dental resin impression method was used with nail polish as an impression material (Kagan et al. 1992; Geisler et al. 2000). Impressions were observed on glass slides using a Zeiss LSM710 NLO two-photon microscope (Mannheim, Germany).

## Additional files

Additional file 1: Supporting information. **Table S1.** List of primers used in this study (F, forward primer; R, reverse primer; q, quantitative real-time PCR). **Figure S1.**The grain width and 1000-grian weight of the WT, over-expression lines and RNAi line.Values are means, and error bars represent the SD from three independent experiments. Asterisks indicate a significant difference between WT and transgenic lines (t-test; **, *P* < 0.01). **Figure S2.**The percentages of water loss of detached leaves from WT and transgenic lines.Values are means, and error bars represent the SD from three independent experiments. (DOCX 825 kb)

## Abbreviations

BCECF-AM: 2’,7’-bis-(2-carboxyethyl)-5-(and-6)-carboxyfluorescein-acetoxymethyl
BCL-2: B-cell lymphoma-2
GFP: Green fluorescent protein
MPK6: Mitogen-activated protein kinase 6
PCD: Programmed cell death
qRT-PCR: Real time quantitative polymerase chain reaction
ROS: Reactive oxygen species
SOS: Salt-overly-sensitive
VPE: Vacuolar processing enzymes
