# Supplementary material for: Suppression of OsVPE3 Enhances Salt Tolerance by Attenuating Vacuole Rupture during Programmed Cell Death and Affects Stomata Development in Rice
Source: Rice (N Y). 2016 Nov 29;9:65. doi: 10.1186/s12284-016-0138-x (PMC5128010; doi:10.1186/s12284-016-0138-x)
Supplement: Additional file 1: — Supporting information. Table S1. List of primers used in this study (F, forward primer; R, reverse primer; q, quantitative real-time PCR). Figure S1.The grain width and 1000-grian weight of the WT, over-expression lines and RNAi line.Values are means, and error bars represent the SD from three independent experiments. Asterisks indicate a significant difference between WT and transgenic lines (t-test; **, P < 0.01). Figure S2.The percentages of water loss of detached leaves from WT and transgenic lines.Values are means, and error bars represent the SD from three independent experiments. (DOCX 825 kb) [file 12284_2016_138_MOESM1_ESM.docx]

**SUPPORTING INFORMATION**

**Table S1****.** List of primers used in this study（F, forward primer; R, reverse primer; q, quantitative real-time PCR）

| Primer name | Sequence (5’-3’) |
| --- | --- |
| *OsVPE3*-OE-F | CACCATGGCGGCGCGGTGGTG |
| *OsVPE3*-OE-R | AGCACTGTACCCCTGCACCAGCGA |
| *OsVPE3*-RNAi-F | CACCAGGGAGGGCTAAAGGAGGA |
| *OsVPE3-*RNAi-R | GCATTTGCAGGGTCGAAAC |
| *OsVPE1*-F | CACCATGGCTGCGCGGTGCTG |
| *OsVPE1*-R | AGCACTGTGTCCTCC |
| q*OsVPE1*-F | GTGCTGGGTATGGGGCTTC |
| q*OsVPE1*-R | TCCTTCACTCCTCCCTTCTGC |
| q*OsVPE2*-F | CGGCTCCAACGGCTACTACAAC |
| q*OsVPE2*-R | TCGGGACCCCAGCATAGACA |
| q*OsVPE3*-F | TCAGCCACTCGTTGATGATTG |
| q*OsVPE3*-R | CTTCCAAAGGGATAGACGCA |
| q*OsVPE4*-F | CAAAGGCAGCCACTCCTACAC |
| q*OsVPE4*-R | GCACTCCCAGTCCTCAACCAG |
| q*OsUBQ5*-F | ACCACTTCGACCGCCACTACT |
| q*OsUBQ5*-R | ACGCCTAAGCCTGCTGGTT |


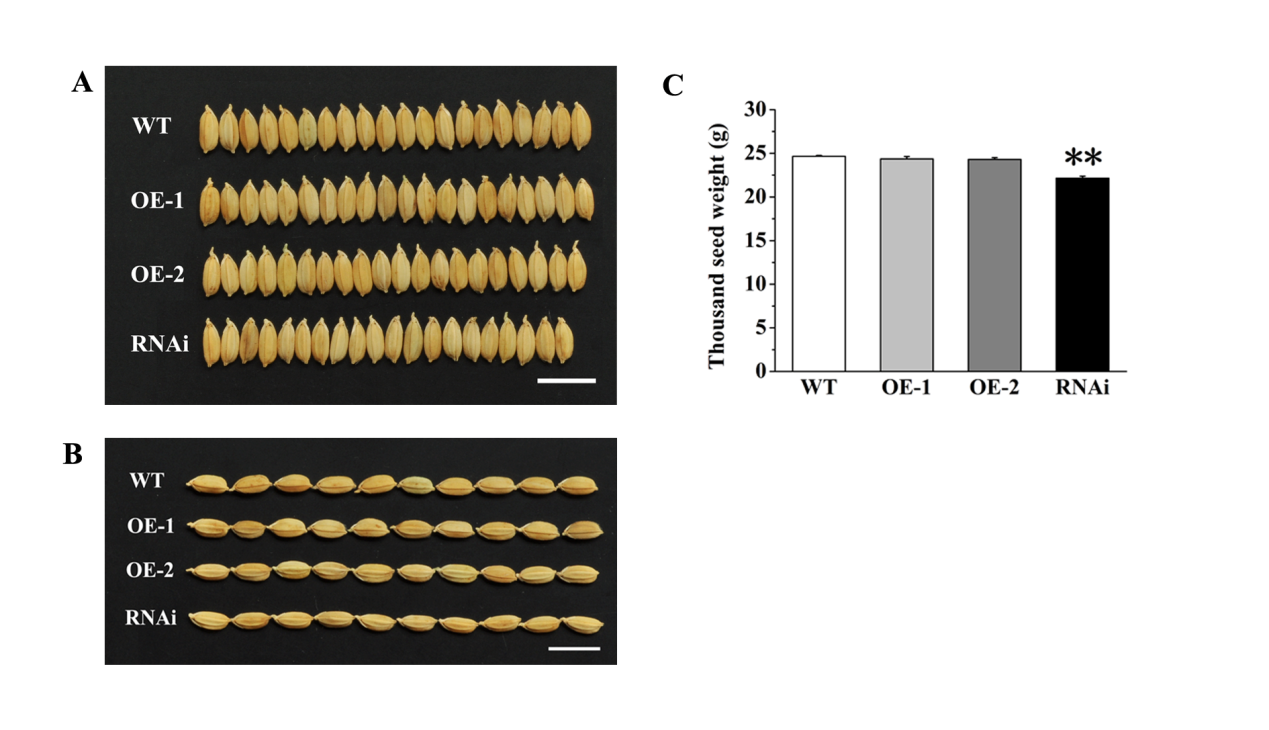


**FigureS1.**The grain width and 1000-grian weight of the WT, over-expression lines and RNAi line.Values are means, and error bars represent the SD from three independent experiments. Asterisks indicate a significant difference between WT and transgenic lines (t-test; **, P < 0.01).


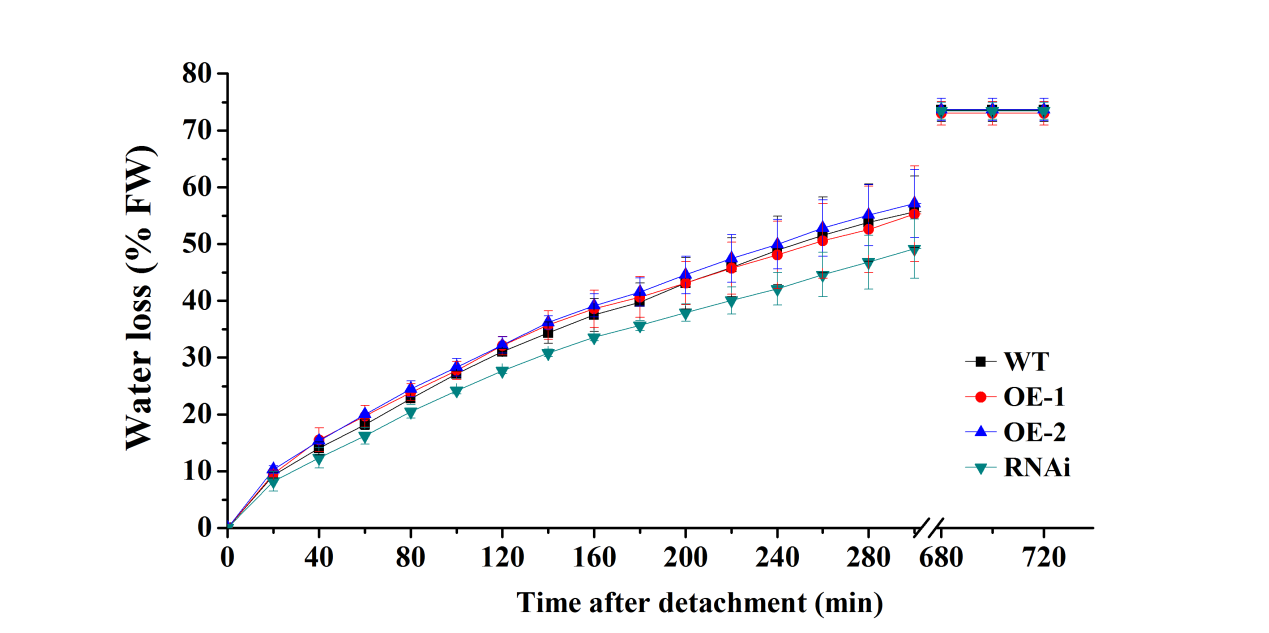


**FigureS2.**The percentages of water loss of detached leaves from WT and transgenic lines.Values are means, and error bars represent the SD from three independent experiments.
